# Fatores de Risco para Infecção do Sítio Cirúrgico Pós Cirurgia Cardíaca Pediátrica

**DOI:** 10.36660/abc.20240015

**Published:** 2024-02-22

**Authors:** Rafael Quaresma Garrido, Cristiane da Cruz Lamas

**Affiliations:** 1 Instituto Nacional de Cardiologia Rio de Janeiro RJ Brasil Instituto Nacional de Cardiologia, Rio de Janeiro, RJ – Brasil; 2 Instituto Nacional de Infectologia Evandro Chagas/FIOCRUZ Rio de Janeiro RJ Brasil Instituto Nacional de Infectologia Evandro Chagas/FIOCRUZ, Rio de Janeiro, RJ – Brasil; 3 Universidade Estácio de Sá/IDOMED Rio de Janeiro RJ Brasil Universidade Estácio de Sá/IDOMED, Rio de Janeiro, RJ – Brasil

**Keywords:** Cardiopatias Congênitas/cirurgia, Infecção de Ferida Cirúrgica, Complicações Pós-Operatórias, Staphylococus Aureus, Síndrome de Imunodeficiência, Escore de RACHS

Infecções após cirurgia cardíaca pediátrica são eventos adversos importantes que podem aumentar a morbidade e a mortalidade nesses pacientes. A maioria dos trabalhos descrevem a incidência de infecção entre 0,5 e 8%, mas alguns estudos em países de renda baixa a média, citam taxas de até 48%.^1-3^ Os principais microrganismos envolvidos são *Staphylococcus aureus*, estafilococos coagulase -negativa e ocasionalmente Gram negativos hospitalares. Em países de renda alta observa-se o predomínio do Gram positivos de pele, enquanto em países de renda baixa a média ocorre um aumento da frequência dos Gram negativos hospitalares.^1-3^

Há divergências entre os fatores de risco para infecção de cirurgia cardíaca pediátrica, pois são poucos os trabalhos contemplando essa população. Em relação ao pré-operatório ou condições inerentes ao paciente, temos como fatores de risco crianças mais jovens, principalmente com idade abaixo de 12 meses, a presença de imunodeficiências, períodos prolongados de internação pré-operatória e o uso prévio de antimicrobianos (ambos por alterarem a microbiota colonizadora) e a desnutrição. Em relação a pré e pós operatório, temos como fatores de risco a inadequação da antibioticoprofilaxia, quebras da técnica asséptica, tempo elevado de *bypass* cardiopulmonar(>105 minutos) e clampeamento da aorta (>85 minutos), sangramento excessivo nas primeiras 24h, transfusão sanguínea, reabordagem por sangramento, tórax aberto no pós operatório, ocorrência de infecções nosocomiais (pneumonia e infecções de corrente sanguínea principalmente) e a permanência de dispositivos invasivos como drenos e fios de marcapasso.^1-6^

Os autores do artigo foco deste mini editorial obtiveram três fatores de risco na análise multivariada de seu estudo de centro único (INCOR), caso-controle, na cidade de São Paulo, nos anos de 2011 a 2018, em crianças de 0 a 19 anos incompletos: idade menor que 2 anos, síndrome genética e pontuação na escala de RACHS ≥ 3.^7^ Todos estes fatores já foram descritos em estudos publicados na literatura. Tanto a idade precoce, quanto a presença de síndromes genéticas, levam a estados de imunodeficiência. A primeira por imaturidade do sistema imune e a segunda por alterações na imunidade celular, humoral e na capacidade de fagocitose. A condição genética mais encontrada foi a síndrome de Down. O terceiro fator preditivo foi a pontuação elevada na escala de RACHS, que é um modelo preditor de mortalidade baseado na complexidade dos procedimentos paliativos e corretivos das cirurgias congênitas.^7^ Escores altos estão relacionados a procedimentos complexos, que levam a tempos cirúrgicos prolongados causando um insulto inflamatório maior no pós-operatório.^8^

Foi descrito pelos autores que valores mais elevados de proteína C reativa (PCR-T) no grupo controle foram significantes na análise multivariada, sendo um fator protetor.^7^ A razão para tal efeito protetor seria atividade opsonizante da PCR-T para microrganismos como o *Staphylococcus aureus.* É bem descrito o aumento da PCR-T, assim como outros marcadores inflamatórios (procalcitonina), após cirurgia cardíaca. Estudos prospectivos observacionais que abordaram a questão mostraram que houve pico da PCR-T cerca de 48 a 72 hs. após o procedimento, mas que não foi possível estabelecer valores que diferenciassem entre infecção e o estado inflamatório neste período.^4,9-11^ Mais estudos são necessários para definir o papel desses marcadores no pós-operatório de cirurgia cardíaca pediátrica.

A fim de reduzir essas infecções alguns autores sugerem a abordagem sistemática das crianças que serão submetidas a cirurgia. É proposto a adoção de *bundles*, ou conjunto de medidas, que contenham aquelas mais relevantes para prevenção de infecção; são estas: otimização do estado nutricional no pré-operatório, uso correto da antibioticoprofilaxia, o preparo da pele com clorexidina, a troca de luvas pelo cirurgião após esternotomia e antes do fechamento do esterno, o controle glicêmico no pré, intra e pós operatório, a cobertura estéril da ferida operatória por 48 hs, e, por fim, a redução de outras infecções nosocomiais.^2,3,12^

A compreensão desses fatores de risco e a adoção das medidas preventivas é essencial para melhorar os resultados em cirurgia cardíaca pediátrica e reduzir a incidência de complicações infecciosas.
