# Supplementary material for: Fatores de Risco para Infecção do Sítio Cirúrgico Pós Cirurgia Cardíaca Pediátrica
Source: Arq Bras Cardiol. 2024 Feb 22;120(12):e20240015. [Article in Portuguese] doi: 10.36660/abc.20240015 (PMC11098576; doi:10.36660/abc.20240015)
Supplement: Translation

# Translation: Risk Factors for Surgical Site Infection after Pediatric Cardiac Surgery

**Authors:** Rafael Quaresma Garrido, Cristiane da Cruz Lamas

**Affiliations:** 1 Rio de Janeiro RJ Brazil Instituto Nacional de Cardiologia, Rio de Janeiro, RJ – Brazil; 2 Rio de Janeiro RJ Brazil Instituto Nacional de Infectologia Evandro Chagas/FIOCRUZ, Rio de Janeiro, RJ – Brazil; 3 Rio de Janeiro RJ Brazil Universidade Estácio de Sá/IDOMED, Rio de Janeiro, RJ – Brazil

**Keywords:** Heart Defects, Congenital/surgery, Surgical Wound Infection, Postoperative Complications, Staphylococus Aureus, Immunologic Deficiency Syndrome, RACHS Score

Infections after pediatric cardiac surgery are important adverse events that can increase morbidity and mortality in these patients. Most studies describe the incidence of infection as between 0.5 and 8%, but some studies in low- to middle-income countries report rates of up to 48%.^1-3^ The main microorganisms involved are *Staphylococcus aureus*, coagulase-negative staphylococci, and occasionally hospital-acquired Gram negatives. In high-income countries, there is a predominance of skin-colonizing Gram-positives, while in low- to middle-income countries, there is an increase in the frequency of hospital-acquired Gram-negatives. ^1-3^

There are divergences between the risk factors for infection in pediatric cardiac surgery, as there are few studies dedicated to this population. In regard to pre-operative conditions or those inherent to the patient, we have as risk factors being a younger child, especially those under 12 months of age, the presence of immunodeficiencies, prolonged periods of pre-operative hospitalization and previous use of antimicrobials (the latter two conditions because they alter the colonizing microbiota) and malnutrition. Pre and post operative risk factors are the inadequacy of antibiotic prophylaxis, breaches of aseptic technique, prolonged cardiopulmonary bypass time (>105 minutes) and aortic clamping time (>85 minutes), excessive bleeding in the first 24 hours, blood transfusion, early chest reexploration due to operative bleeding, occurrence of nosocomial infections (pneumonia and bloodstream infections mainly) and the presence of invasive devices such as drains and pacemaker wires.^1-6^

The article in discussion in this short editorial reported three risk factors in the multivariate analysis of their single-center study (INCOR), case-control, in the city of São Paulo, from 2011 to 2018, in children aged 0 to 19 incomplete years: age less than 2 years, the presence of a genetic syndrome and a RACHS scale score ≥ 3.^7^ All these factors have already been described in studies published in the literature. Both early age and the presence of genetic syndromes lead to immunodeficiency states. The first is due to the immaturity of the immune system, and the second due to changes in cellular and humoral immunity and the capacity for phagocytosis. The most common genetic condition was Down syndrome. The third predictive factor was a high score on the RACHS scale, which is a mortality prediction model based on the complexity of palliative and corrective procedures in congenital surgeries.^7^ High scores are related to complex procedures, which lead to prolonged surgical times, causing a greater inflammatory insult in the post-operative period.^8^

The authors report that higher values of C-reactive protein (CRP) in the control group were significant in the multivariate analysis, being a protective factor.^7^ The reason for such a protective effect would be the opsonizing activity of CRP for microorganisms such as *Staphylococcus aureus*. The increase in CRP, as well as other inflammatory markers (procalcitonin), after cardiac surgery is well described. Prospective observational studies that addressed the issue showed that CRP peaks approximately 48 to 72 hours after the procedure, but it was not possible to establish values that differentiated between infection and the inflammatory state during this period.^4,9-11^ More studies are needed to define the role of these markers in the post-operative period of pediatric cardiac surgery.

In order to reduce these infections, some authors suggest a systematic approach to children who undergo surgery. Bundles, or specific sets of measures, are proposed; these contain the most relevant measures for preventing infection. These bundles involve optimization of nutritional status pre-operatively, correct use of antibiotic prophylaxis, preparation of the skin with chlorhexidine, change of gloves by the surgeon after sternotomy and before closing the sternum, glycemic control pre-, intra- and post-operatively, sterile coverage of the surgical wound for 48 hours, and, finally, the reduction of other nosocomial infections.^2,3,12^

Understanding these risk factors and adopting preventive measures is essential to improve results in pediatric cardiac surgery and to reduce the incidence of infectious complications.
